# Metabolites in the Tumor Microenvironment Reprogram Functions of Immune Effector Cells Through Epigenetic Modifications

**DOI:** 10.3389/fimmu.2021.641883

**Published:** 2021-04-13

**Authors:** Yijia Li, Yangzhe Wu, Yi Hu

**Affiliations:** ^1^ Zhuhai Institute of Translational Medicine, Zhuhai People’s Hospital (Zhuhai Hospital Affiliated With Jinan University), Zhuhai, China; ^2^ Biomedical Translational Research Institute, Jinan University, Guangzhou, China; ^3^ Microbiology and Immunology Department, School of Medicine, Jinan University, Guangzhou, China

**Keywords:** tumor microenvironment, metabolites, immune cell reprogramming, epigenetic modifications, anti-tumor immunity

## Abstract

Cellular metabolism of both cancer and immune cells in the acidic, hypoxic, and nutrient-depleted tumor microenvironment (TME) has attracted increasing attention in recent years. Accumulating evidence has shown that cancer cells in TME could outcompete immune cells for nutrients and at the same time, producing inhibitory products that suppress immune effector cell functions. Recent progress revealed that metabolites in the TME could dysregulate gene expression patterns in the differentiation, proliferation, and activation of immune effector cells by interfering with the epigenetic programs and signal transduction networks. Nevertheless, encouraging studies indicated that metabolic plasticity and heterogeneity between cancer and immune effector cells could provide us the opportunity to discover and target the metabolic vulnerabilities of cancer cells while potentiating the anti-tumor functions of immune effector cells. In this review, we will discuss the metabolic impacts on the immune effector cells in TME and explore the therapeutic opportunities for metabolically enhanced immunotherapy.

## Introduction

Cancer is one of the leading causes of death globally. Although numerous efforts and progress have been made, curing cancer is still a far-reaching goal thus far. Traditional cancer treatment strategies include surgery, radiation, and chemotherapy. However, other than the common side-effects, studies have shown dire consequences of these strategies, such as higher tumorigenic, metastatic rates, the production of cancer stem cells, the induction of drug resistance, and accelerated aging, etc. (1, 2). Therefore, in recent years, immune cell therapies have attracted increasing attention as one of the best alternative treatment strategies for cancer (3–5). Although promising outcomes have been achieved, such as the application of Chimeric Antigen Receptor (CAR)-T therapy in treating B cell lymphoma (6–8), researchers made limited progress on using immune cell therapy to treat solid tumors. At the same time, our group also developed a new immune cell strategy for cancer immunotherapy, we applied allogeneic Vγ9Vδ2 γδ T cells that originated from healthy donors to treat solid tumors (9, 10) and found that patients respond to this therapy differently. This suggested that whether adoptively transferred immune cells can function properly in the tumor microenvironment (TME) is the key to successful clinical therapy. Commonly, the negative efficacy can be partly attributed to the complexity and the immunosuppressive nature of the tumor microenvironments (TME). Therefore, to design better immune cell therapies in cancer treatment, scientists need a clear understanding of the multiple aspects that compose and help shape the complexity of TME. It is well known that cancer cells can thrive and meanwhile evade immune cell recognition through “immunoediting” in the TME. Importantly, the acidic, hypoxic, and nutrient-deficient TME provides a competitive advantage to cancer cells to outcompete immune cells (11, 12).

Therefore, an insightful understanding of how TME edits or suppresses infiltrated immune cells is crucial for developing an optimal immune cell strategy to treat solid tumors. Till now, the overview landscape for tumor infiltrated immune cells has been largely established and can be briefly classified into two functional populations, immune suppressive and effector cell. The typical infiltrated suppressive cell includes regulatory T/B cell (T_reg_/B_reg_), myeloid-derived suppressor cell (MDSC), M2-like Macrophage, etc., which had been reviewed previously (13–16). As for as infiltrated immune effector cell is concerned, CD8^+^ cytotoxic T cell, Th1, NK, and γδ T cell are representative populations and have been extensively investigated. In this review, we will mainly focus on current literature of the influence of TME on the immune effector cell, particularly, we are trying to sketch how TME uses metabolites to reprogram infiltrated immune effector cells to accomplish immune escape. Under such context, how cancer cells take advantage of the unique microenvironment to conquer immune cells needs to be briefly introduced at the start of this review.

## TME Uniquely Inhibits Anti-Tumor Immunity

### TME is a Low pH Environment

Malignant cells preferentially use aerobic glycolysis rather than the more energy-efficient mitochondrial phosphorylation as the energy source, known as the “Warburg effect” (17). The end-product of the glycolytic pathway is lactate, the main contributor to the acidic nature of the TME. Studies indicated that lactate could be further used by cancer cells to fuel their metabolism, drive M2 macrophage polarization (18), and severely inhibit the effector functions of cytotoxic, helper T cells (Th1/2, Tc), and natural killer cells in the TME (12, 19–22). Moreover, lactate supports the metabolic need for tumor infiltrated Treg (23, 24), which suppresses effector T cell functions in TME.

### Hypoxia is a Hallmark of TME

**Figure 1:**
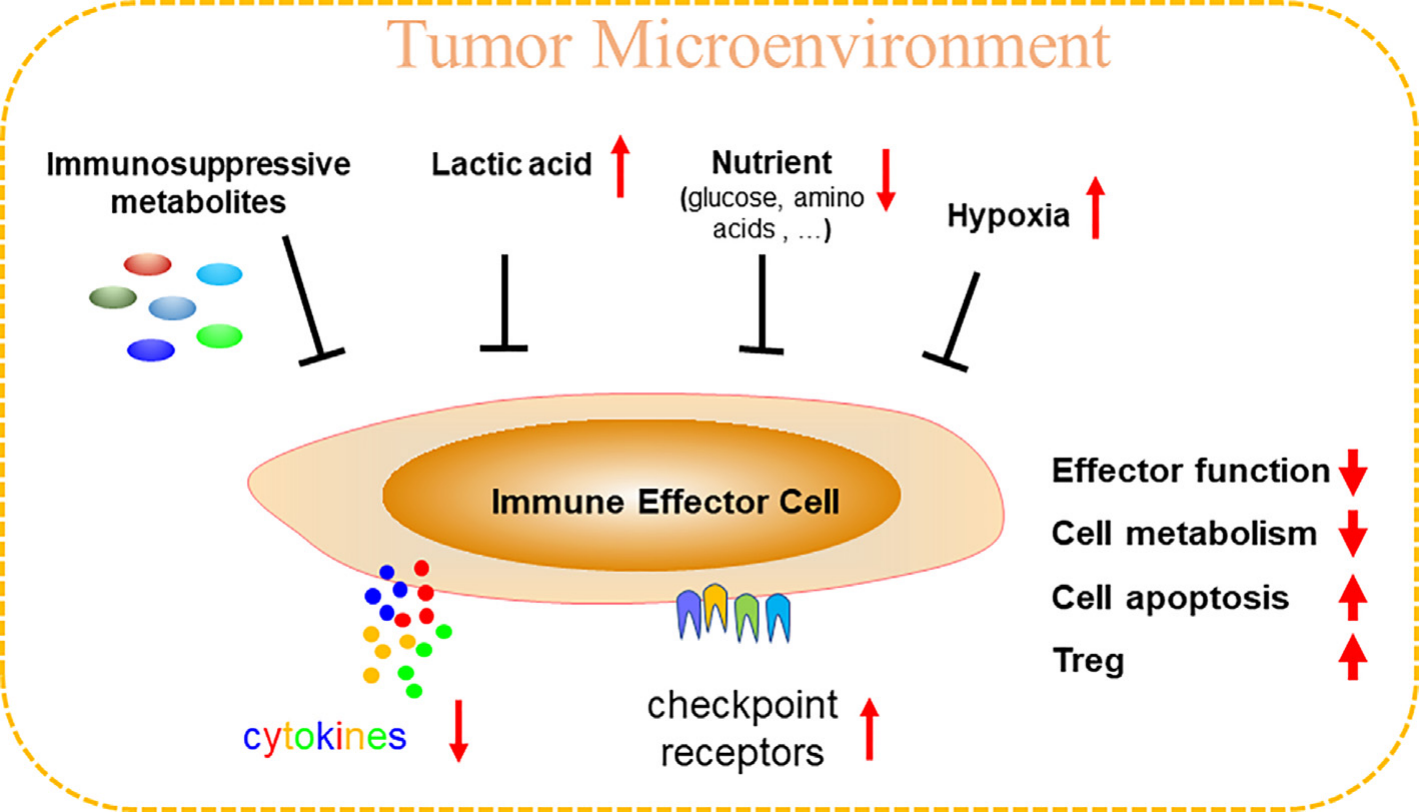
Tumor microenvironment (TME) can specifically inhibit anti-tumor immunity. TME is a hypoxia environment accompanying by high lactic acid and nutritional deficiency, thus produces abundant and various immunosuppressive metabolites. Immune effector cells (cytotoxic T, Th1, NK, γδ T, etc.) in TME are therefore comprehensively inhibited or disrupted, including reducing cytokines release, upregulations of checkpoint receptors, cell cycle arrest, cell metabolism disturbance, increased cell apoptosis, and unfortunately, TME could recruit immunosuppressive immune cells like Treg to reinforce the immunosuppressive microenvironment.

The uncontrolled cancer cell proliferation inevitably leads to increased oxygen consumption, together with the malformation of the tumor vascular systems, leads to insufficient oxygen supply in the TME, also called hypoxic conditions (25). Hypoxia would further induce Hypoxia-inducible factor-1 alpha (HIF-1α) expression, facilitating the cancer cell adaptation in the oxygen-deficient TME. HIF-1α expression promotes cancer glycolysis and evasion of immunosurveillance, at the same time, tampering with anti-tumor immunity directly by inhibiting NKG2D expression in NK cells (26, 27), reducing CD4^+^ effector T cell differentiation (28), promoting regulatory T cell differentiation and activity, elevating checkpoint molecule expression (29, 30), as well as inducing T cell apoptosis (31). Moreover, Hypoxia could indirectly drive immunosuppressive metabolites production to support the rapid proliferation of cancer cells (32). Interestingly, the study also demonstrated *in vitro* hypoxic culture conditions would enhance the anti-tumoral functions of CD8^+^ T cells (33), and research further suggested different T cell subpopulations could respond to hypoxia quite differently. For example, while human CD8^+^ naïve and central memory T cells were impaired, the functions (proliferation, viability, and cytotoxicity) of effector memory CD8^+^ T cells could be enhanced in the context of hypoxic conditions (34). These works showed that hypoxia plays various important roles in regulating T cell function (35), and hypoxia-inducible factors (HIF) are involved in mediating the metabolic shift from aerobic respiration to glycolysis as well as enhancing effector function of certain T cell sub-populations in both human and murine (33, 34, 36, 37). Similarly, in mouse CD4^+^ T cells, augmented HIF activity can promote glycolysis and induce the conversion of Treg into IFN-γ^+^ T_H_1-like cells (38–40), however, HIF function in human CD4^+^ T cells remains to be fully addressed. Therefore, a hypoxic condition in TME affects infiltrated immune cells from multiple dimensions. Nevertheless, even though immune effector cells can survive and fulfill functions in hypoxic conditions, functional defects of naive T cell led to failure of its differentiation into the effector T cell, which can eventually compromise the immune balance in the host (**Figure 1**). Additionally, as far as NK is concerned, hypoxia can inhibit the expression of activation-, cytotoxicity-, effector-related molecules of NK cells in both human (41) and murine (42), even though NK cells can still kill target cells *via* antibody‐dependent cellular cytotoxicity (ADCC) (41), which suggested HIF-1α behave differently in NK comparing to αβ T cells. Similar to NK, γδ T cells in the TME of mice model also exhibited-hypoxia induced antitumor repression, and HIF-1α also acted adversely (43, 44).

## Anti-Tumor Immunity of Immune Cells is Disrupted in TME Due to Loss of The Nutritional Battle

There is a constant nutrition battle between cancer and immune cells in TME (**Figure 1**). Nutrients such as glucose, amino acids in the TME are often consumed faster by tumor cells than infiltrated immune cells, which thus stripes the energy source that fuels the effector functions of immune cells (45). The imbalance of energy consumption and metabolite productions in the TME further influences the signal transduction and gene expressions among cells in TME, creating an immunosuppressive environment that further supports tumor growth (11). A few elegant studies done by Pearce’s group demonstrated that lFN-γ production by effector T cell could be dampened in TME due to the loss of aerobic glycolysis in T cells (46). Their follow-up study further indicated that checkpoint blockade antibodies against CTLA-4, PD-1, and PD-L1 could restore T cell glycolysis and lFN-γ production. Ho et al. showed that glycolytic metabolite phosphoenolpyruvate (PEP) sustains calcium and TCR signaling of effector T cells, increasing PEP production could metabolically reprogram tumor-specific T cell and potentiate their anti-tumor response in TME (47). Such reports suggested that interfering metabolites in TME can rebalance the microenvironment to be suitable for anti-tumor immune effect, and eventually benefit outcomes of tumor immunotherapy. It should be also noted here that inhibited glycolytic metabolism of infiltrated CD8^+^ cytotoxic T cells in TME does not mean an absolute disaster, because glycolysis inhibition could enhance the generation of neonatal memory CD8^+^ T cells and antitumor function as well (48, 49). Therefore, the plasticity of infiltrated immune cells should be profoundly understood and be strategically utilized in tumor immunotherapy.

### Tuning Amino Acids in TME Regulates Immune Effector Cell Function

Furthermore, amino acid deprivation in TME poses another metabolic challenge to tumor-infiltrated immune cells. For instance, restricting methionine intake from the diet was claimed to effectively slow down tumor growth in the PDX mice model (50), nonetheless, critically impaired T cell effector functions as well as T_H_17 differentiation (51, 52). T cell responds to antigenic challenge in the TME by upregulating its amino acid intake to fuel its effector function. This is a process coordinated by the T cell antigen receptor (TCR) and determines T cell differentiation (53). For instance, glutamine is an important amino acid for the proper development of both cancer cells and tumor-infiltrated immune cells. Glutamine regulates mTOR activation (54) and O-GlcNAcylation (55) in effector T cells, which are keys stages for T cell development and function. It is also the main carbon source for the oncometabolite 2-hydroxyglutarate, which regulates the functions and differentiation of effector T cells (56). Nevertheless, conflicting results have been shown on whether limiting glutamine metabolism could strengthen anti-tumor functions of effector T cells (57–59). Recent studies have demonstrated the essential roles of other amino acids such as Arginine (60–62), leucine (63), serine (64) in modulating T cell proliferation and anti-tumor efficacy. However, due to the complexity of tumor infrastructure, the distribution and variation of these nutrients within TME still await further elucidation.

Since there is metabolic plasticity in immune cells, it might be plausible to metabolically target cancer and immune cells (glutamine, methionine, etc.) to enhance the immune effector cell function while inhibiting cancer progression. In this context, it is an urgent need to better understand the roles of different TME metabolites and their related metabolic pathways in TME.

### Lipid Metabolism Regulates Immune Effector Cell Function in TME

Lipid metabolism is mainly comprised of fatty acid and cholesterol metabolism (65). Lipid metabolism could regulate tumor-infiltrated immune cells, for example, modulate Treg functions through influencing mitochondria integrity (66). Effector T cell activation and proliferation require accelerated lipid synthesis and cholesterol uptake since both are crucial components of the cellular membrane. These processes are mediated by transcription factor sterol regulatory element-binding proteins (SREBPs). The lack of functional SREBPs signal in CD8+ T cells leads to attenuated clonal expansion and effector functions (67); as a contrast, increasing cholesterol content in the plasma membrane can enhance CD8+ T cell anti-tumor functions (68). This could be interpreted by a previous report that memory CD8+ T cells rely on cell intrinsic-lipolysis to synthesize fatty acid whereas effector CD8+ T cell (Teff) obtained fatty acids from the external microenvironment (69). Therefore, lipid metabolism was considered to regulate the balance between Treg and Teff in TME (70). Nevertheless, it also showed that high cholesterol in TME could induce CD8+ T cell exhaustion by overexpressing immune checkpoints, such as PD-1, TIM-3, LAG-3, and 2B4, and increasing endoplasmic reticulum (ER) stress (71). Such discrepancy might attribute to the heterogeneity of TME in different cancer types, thus, albeit important for effector T cell metabolism and function, targeting lipid or cholesterol metabolism to potentiate anti-tumor response requires further investigation.

Though metabolic pathways such as glycolysis and oxidative phosphorylation (OXPHOS) are seemingly critical for the thriving of both cancer and infiltrated immune cells, considerable metabolic heterogeneity and plasticity allow us to differentiate the two populations. The advent of single-cell sequencing technologies enables metabolic profiling of TME at a single-cell resolution. For instance, a previous single-cell study revealed a metabolic heterogeneity among cells in TME, with mitochondrial programs being the most distinguishing factor in shaping this heterogeneity in malignant cells and immune cells (72). Metabolites and immunosuppressive characteristics and cellular networks in TME also help shape the metabolic phenotypes and functions of immune cells (**Figure 1**). Therefore, discerning and understanding the diverse metabolic requirements of infiltrated immune cells that work concertedly against cancer cells enable researchers to selectively modulate immune cell functions (73). The knowledge on the minute discrepancy in metabolic dependency between cancer and immune cells provides opportunities for uncovering new therapeutic targets.

## TME Epigenetically Regulates Immune Effector Cell Functions

“Epi”, a prefix from Greek, literally means “upon, over”, thus epigenetics is the research focus on sets of instructions directed upon the genome, which is composed of chromosomes. Epigenetics studies focus on understanding the heritable changes in gene expressions that do not involve DNA sequence alteration (74). DNA sequences and histone proteins form nucleosomes, the building blocks of chromosomes. Histones provide structural support to help organize and condense DNA. The epigenetic instructions on the genome are sets of chemical modifications, such as methylation, acetylation, etc. made directly to the DNA bases or histone proteins that wrap around them. Different from genetic coding, epigenetic modifications are reversible and dynamic, allowing changes made as the needs of the cells shift. The existence of epigenome allows the fine-tuning of gene expressions in cells. Normally, epigenetic modifications on the genome are a routine occurrence that maintains the healthy balance of the body by instructing the body to turn “on” or “off” certain genes completely as well as slightly “up” or “down” as required. Therefore, it plays critical roles from determining cell fate to directing cellular functions. Nevertheless, dysregulated epigenetic modifications are common in cancer and other diseases (75–78). Drugs that target cancer cell epigenome also achieve positive outcomes (79–82). Studies in recent years also demonstrated the critical role of epigenetic modifications in immune cell functions (83–86). Progress has been made on developing epigenetic immunotherapy for cancer treatments (85, 87). Therefore, more insightful elucidation of epigenetic regulations of both immune cell function or dysfunction in the TME could inevitably help design more effective immunotherapeutic strategies for cancer.

**Figure 2:**
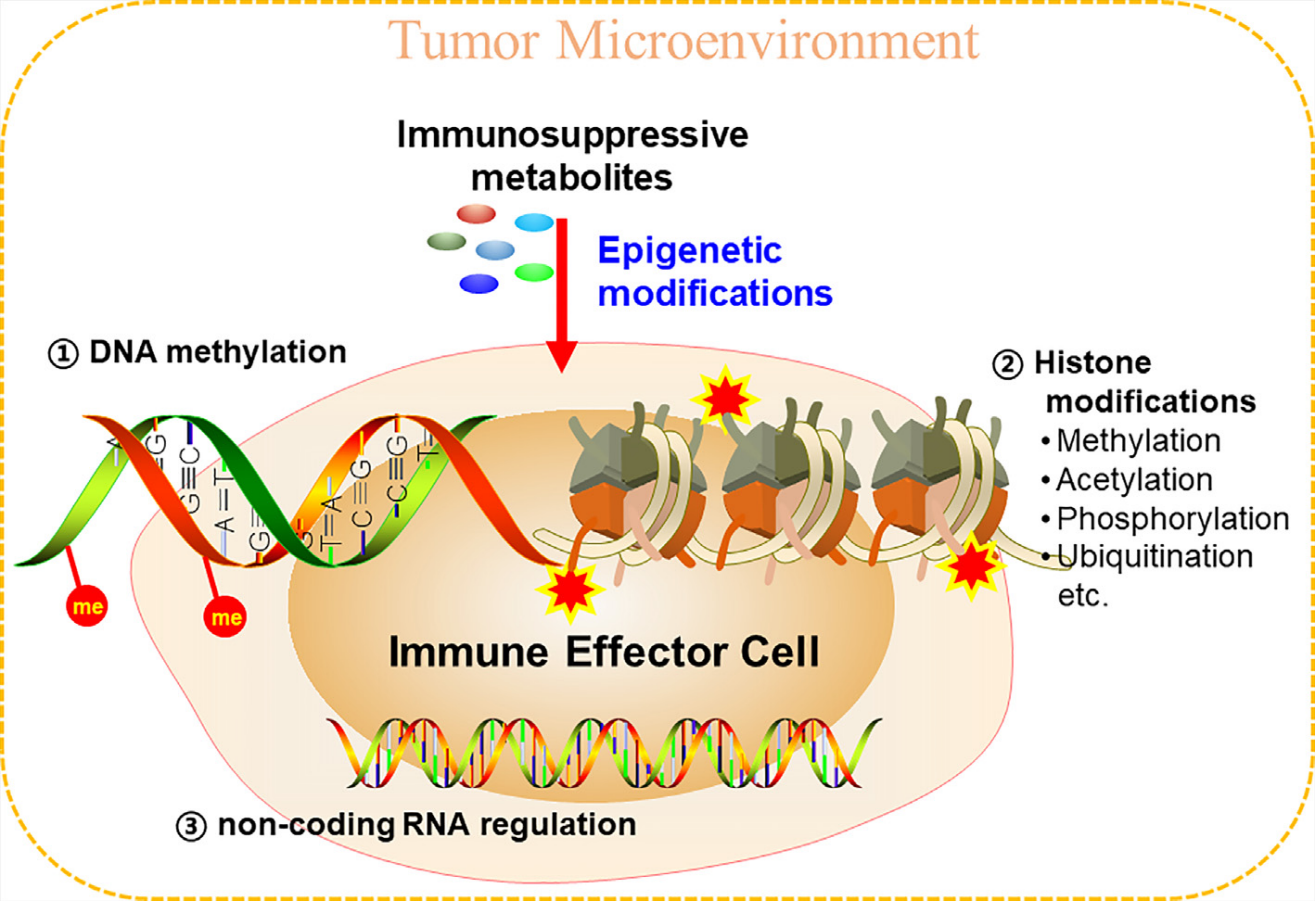
Metabolites in TME could epigenetically reprogram immune cells to inhibit anti-tumor immunity. Epigenetic modifications mainly include three aspects, DNA methylation, histone modifications, and non-coding RNA regulations.

As for epigenetic modifications, there are at least three epigenetic mechanisms that are under intensive investigation, which include: DNA methylation, histone modifications, and non-coding RNA (ncRNA)-associated gene silencing. ncRNA-associated gene silencing is an emerging field that deserves its own comprehensive review (88, 89). Therefore, in this review, we only focused on illustrating the epigenetic modifications of DNA and histone proteins in TME (**Figure 2**).

### TME Stress Induces DNA Methylation of Immune Effector Cells

DNA methylation is the earliest discovered and heavily studied epigenetic modification. It is a chemical process that adds a methyl group (–CH_3_) to the DNA thereby modifying the expression and functional status of genes. This process is catalyzed by DNA methyltransferase (DNMT) and uses S-adenosyl methionine (SAM) as the methyl group donor (90–92). In a pan-cancer context, Mitra et al. explored and discovered varying levels of CpG methylation of immune cell-type-specific genes that are related to patient survival (93). A comprehensive retrospective paper emphasized the importance of clarifying the DNA methylation sites for the development of cancer biomarkers (94). Point mutation of NADP (+)-dependent isocitrate dehydrogenases IDH1(R132H), which occur frequently in glioblastoma, acute myeloid leukemias, etc., showed a strong correlation between tumorigenesis and specific DNA hypermethylation signatures (95). Moreover, accumulating studies also revealed DNA methylation of cancer cells can modulate both cancer and infiltrated immune cell functions in TME. By analyzing sequencing datasets from BLURORINT Epigenome Project, Schuyler et al. discovered distinctive trends in methylation patterns of innate and adaptive immune cells in TME, suggesting distinct lineage-specific epigenetic mechanisms in regulating tumor infiltrated immune cells functions (96). Specific DNA methylation alterations in the circulating immune cells of cancer patients have been observed in head and neck squamous cell carcinoma (HNSCC) (97), ovarian (97, 98), colorectal (99), hepatocellular carcinoma (HCC) (100), and breast cancer (101). Due to their ability to reactivate genes such as tumor suppressors and further elicit immunity towards tumor cells, the development of DNA methylation inhibitors together with immunotherapies, present new cancer treatment opportunities (102).

### TME Stress-Induced Histone Modifications of Immune Effector Cells Remain Largely Unclear

Covalent post-translational modification (PTM) modifications of histone, including acetylation, methylation, phosphorylation, ubiquitylation, and sumoylation, etc., impacting gene expressions by changing chromatin structures, making it either accessible (euchromatin) or inaccessible (heterochromatin) for gene transcriptions (103, 104). Among these epigenetic modifications on histones, acetylation and methylation gained the most attention. Histone acetylation is the addition of an acetyl group to the lysine residues at histone tails. This reaction is catalyzed by histone acetyltransferases and utilizes acetyl CoA as the acetyl group donor. Upon acetylation, the overall charge on histone tails changes from positive to neutral, weakening the interaction between DNA and histone, therefore facilitating gene transcription. On the other hand, histone deacetylation removes the acetyl group from lysine residues of histone tails, making the chromatin highly condensed and inaccessible for transcription. Thus, the balance between euchromatin and heterochromatin could be tightly regulated by histone acetylation and deacetylation (105, 106). Nonetheless, studies showed that histone acetylation/deacetylation status were dysregulated in cancer development (107, 108), such as cervical cancer (109), breast cancer (110), leukemia (108), and non-small cell lung cancer (111, 112). Like histone acetylation, methylation at the histone tails also regulates gene expression (113, 114). Histone methylation takes place at both arginine and lysine residues at histone tails and comes in three different flavors-monomethylated, dimethylated, and trimethylated. Dysregulation of histone methylation has been shown in causing premature aging and cancers (115), such as colorectal cancer (116, 117), glioblastoma (118), and prostate cancer (119). However, how histone of immune effector cells is modified in TME remains to be further investigated, although Silva-Santos’ group investigated the histone methylation patterns and their effect on transcription factors for γδ T cell differentiations in TME of mice model (120). Notably, different inhibitors for histone deacetylase could lead to either suppressed (121) or enhanced (122) human γδ T cell antitumor activity. Thus, histone modification in immune effector cells shall be an interesting research field of antitumor immunity.

## TME Metabolites Epigenetically Reprogram Both Innate and Adaptive Immune Effector Cells

The immunosuppressive nature of TME, mediated by direct comprehensive cell-cell contact and soluble factors such as metabolites, results in alterations in gene expressions in infiltrated immune cells that are partly driven by epigenetic programs. Although extensive efforts have been made on analyzing the histone and DNA epigenetic modifications of cancer cells, little is known about the mechanisms of epigenetic dysregulation of immune cells in the tumor niche (123, 124). Recent findings indicated that immune cells, especially tumor infiltrated ones, show metabolic reprogramming on their differentiation and effector functions. Ovarian cancers-imposed glucose restriction on tumor infiltrated T cells and dampened their function through epigenetically dysregulating histone methylation patterns (125). It’s increasingly considered that both the innate and adaptive arms of the immune network in TME are epigenetically regulated by TME metabolites (e.g., glucose, glutamine, lactate, αKG, 2-HG, etc.).

In the innate arm of the immunity, studies showed that the lineage commitment of myeloid and lymphoid lineage cells is regulated by DNA methylation (126–128). In the myeloid lineage, epigenetic modifiers, including Tet methylcytosine dioxygenase 2 (TET2), isocitrate dehydrogenase 1 (IDH1), IDH2, enhancer of zeste homologue 2 (EZH2) are mutated and lead to defects in DNA and/or histone epigenetic modifications in several myeloid malignancies, such as chronic myeloid leukemia (CML) and acute myeloid leukemia (AML) (129, 130). Zinc Finger E-Box Binding Homeobox 1 (ZEB1), a transcription factor that acts as a tumor suppressor in T-cell acute lymphoblastic leukemia (T-ALL), is repressed due to histone deacetylation and chromatin condensation at its promoter (131).

In the adaptive arm of the immunity, Bian et al. found that by manipulating methionine metabolism in TME, tumor cells lower histone di-methylation at lysine 79 of histone H3 (H3K79me2) in CD8+ T cells, leading to low effector gene expression thus impaired effector T cell immunity. Furthermore, inhibition of the specific and sole methyltransferase for H3K79: DOT1 of CD8+ T cells both *in vitro* and in mice led to the loss of H3K79me2 thus impaired cytotoxicity of CD8+ T cells, which supported their observations in TME (51). Methionine has also been shown to play an essential role in Th17 differentiation and function by regulating histone methylation (52). 2-hydroxyglutarate (2-HG), an oncometabolite caused by IDH mutations that frequently occur in gliomas and acute myeloid leukemia, led to genome-wide histone and DNA methylation alterations (132). S-2-hydroxyglutarate (S-2-HG) in TME could mediate CD8+ T cell differentiation by modulating DNA and histone demethylation status in mice (56). A recent study also indicated that the loss of 2-HG production directly reduced methylation of the Foxp3 gene locus, increasing Fox3 expression, thus reprograms T_H_17 differentiation towards Treg cells (133). Moreover, low glucose availability in TME restricts acetyl-CoA level, the acetyl group donor for histone acetylation (134), and Qiu et al. demonstrated that acetate supplementation rescued CD8+ T cell effector function in a glucose restricted environment by promoting histone acetylation and chromatin accessibility thus promoting IFN-γ production of T cells in TME (135). Besides glucose restriction, glutamine deprivation resulted in the differentiation of immunosuppressive regulatory T (Treg) cells from naive CD4+ T cells due to the loss of α-ketoglutarate (αKG), the glutamine-derived metabolite that is needed for DNA demethylation and regulates CD4+ T cell T_H_1 differentiation. Nevertheless, the addition of αKG analog could shift the differentiation towards that of a T_H_1 phenotype (136). Therefore, although the underlying molecular mechanisms on how TME metabolites serve as activators or inhibitors for epigenetic modifications in immune cells need to be further elucidated, manipulation of metabolic conditions of T cells, particularly effector T cells would provide a potential alternative strategy in the application of T cell-based immunotherapy.

## A New Frontier of Conditioning Metabolism to Enhance Immune Effector Cell Functions in Immunotherapies

Recent advances on epigenetic modification strategies in cancer treatment provide us mechanistic insights into the interplay of immune and tumor cells with their environmental cues (80, 87). DNA methylation inhibitors alone or coupled with other inhibitors to target the epigenetic processes, such as histone deacetylases, methylases, and demethylases, are becoming important treatment regimens in certain cancers, especially hematological malignancies. The epigenetic reprogramming of TME in combination with immunotherapies opens a new therapeutic window for more effective cancer therapies (102). Epigenetic therapies that coupled epigenetic immune modulation with immune therapy priming achieve satisfying preclinical and clinical results in various gastrointestinal cancers (117, 137). Combining DNA-demethylating agents with histone deacetylase inhibitors (HDACis) in non-small-cell lung cancer (NSCLC) treatment regimen reversed tumor evasion and led to robust T cell anti-tumor response (138). Zou group demonstrated DNA methylation by enzyme DNMT1 and histone H3 lysine 27 trimethylation (H3K27me3) by enzyme EZH2 in tumor led to epigenetic silencing of T helper 1 (T_H_1) type chemokine, and subsequent undermined effector T cell trafficking to TME. Using epigenetic modulators (5-AZA-dC, GSK126, etc.) to target these two enzymes could reprogram T cells for more effective T cell immunotherapy (85).

Studies showed that the functions of chromatin-modifying enzymes such as histone acetyltransferases, deacetylases, and DNMT strongly depend on metabolic signals such as acetyl-CoA, Nicotinamide adenine dinucleotide (NAD), and SAM in TME, epigenetically modulating CD8+T cells activation and exhaustion (139). Moreover, metabolites in TME could also upregulate immune checkpoint molecule expressions (140, 141) and suppress immune cell activation (142–144), leading to dampened efficacy of the immune therapies (145). Therefore, metabolic conditioning of CD8+ or other immune cell functions in TME might help overcome the current weaknesses of immune cell-based immunotherapies. Recent findings in immune cell metabolic reprogramming indicated the possibilities of clinical metabolic interventions for cancer treatment (12, 146). Metabolic intervention by sodium bicarbonate helps neutralize the lactate acidity in AML, leading to improved efficacy of CD8+T cell immunotherapy (147). Pearce group showed that transient glucose restriction (TGR) in CD8+effector T cell before adoptive transfer metabolically condition effector T cell functions and enhance tumor clearance in mice (148). Additionally, clinical studies on epigenetic therapy for cancer have been previously reviewed (81, 149), showing that targeting epigenetic modifications or regulators in cancer cells would potentiate anti-tumor immune therapy.

## Summary

In this review, we focused on immune effector cells in TME and reviewed literature about how epigenetic modifications, in the form of DNA methylation and histone acetylation/methylation, can be modulated by metabolites and other environmental cues in TME. We also discussed the current advances in using metabolic modifiers to epigenetically enhance the efficacy of immune cell therapy. From this review, one can see that immune effector cells in TME are comprehensively reprogramed to be either exhausted effectors, by-standers, or conspirators of cancer cell escape, and metabolites in TME participate in this ugly job. Nevertheless, opportunities coexist with the crisis, targeting TME metabolites could potentially be a valuable supplement to the application of immune cell-based immunotherapy for cancer.

## Author Contributions

YL, literature research and summary. YW and YH, manuscript writing and revision. All authors contributed to the article and approved the submitted version.

## Funding

YH was supported by the National Natural Science Foundation of China (NO. 82002787); YW was supported by the Natural Science Foundation of Guangdong Province, China (2020A1515010132).

## Conflict of Interest

The authors declare that the research was conducted in the absence of any commercial or financial relationships that could be construed as a potential conflict of interest.
